# The aging immune system in Alzheimer’s and Parkinson’s diseases

**DOI:** 10.1007/s00281-022-00944-6

**Published:** 2022-05-03

**Authors:** Kelsey S. Heavener, Elizabeth M. Bradshaw

**Affiliations:** Department of Neurology, Columbia University Irving Medical Center, New York, NY 10032 USA

**Keywords:** Alzheimer’s disease, Parkinson’s disease, Microglia, T cells, Immunosenescence

## Abstract

The neurodegenerative diseases Alzheimer’s disease (AD) and Parkinson’s disease (PD) both have a myriad of risk factors including genetics, environmental exposures, and lifestyle. However, aging is the strongest risk factor for both diseases. Aging also profoundly influences the immune system, with immunosenescence perhaps the most prominent outcome. Through genetics, mouse models, and pathology, there is a growing appreciation of the role the immune system plays in neurodegenerative diseases. In this review, we explore the intersection of aging and the immune system in AD and PD.

## Introduction

Over the past decade, it has become clear that the immune system plays a central role in neurodegenerative diseases [1, 2]. Parkinson’s disease (PD) and Alzheimer’s disease (AD) are both neurodegenerative diseases with immune and genetic components, but they each have distinct pathological and clinical phenotypes. The study of the innate immune system in these diseases has become a major focus for the field. Despite these significant research efforts, most of the immune processes that have been implicated in AD and PD remain poorly understood. The resident central nervous system (CNS) innate immune cells, microglia, have been the focus of these efforts thus far. However, there is also a vital need to understand the role of infiltrating adaptive immune cells, specifically T cells, that may either maintain healthy neurons in the affected regions of the brain or lead to neuronal loss in disease contexts.

Aging is the largest risk factor for both AD and PD, and importantly also severely impacts the immune system’s fitness [3, 4]. By definition, the predominant form of AD, late-onset AD (LOAD), is a disease of aging as one must be over 65 years of age to be diagnosed with LOAD. In contrast, early-onset Alzheimer’s disease (EOAD) presents before age 65 and occurs in only 5% of cases [5]. The global burden of AD is only expected to grow as the world’s aging population continues to increase. Aging is also a primary risk factor for Parkinson’s disease [6]. The incidence of Parkinsonism (an umbrella term that refers to a group of disorders that cause movement disturbances as classically seen in Parkinson’s disease such as tremors, bradykinesia, and rigidity) increases in the elderly and becomes very common in populations over age 65 [7]. In addition, age of onset for PD affects disease progression, with those developing late-onset disease exhibiting more severe and rapid disease progression [8, 9]. Patients who were older at the time of disease onset exhibited more severe bradykinesia and rigidity and were more likely to have a balance disorder [8]. Hence, understanding the contribution of aging to these diseases is critical for segregating subsets of patients for correct clinical treatments.

## Aging and the immune system

Reduced efficacy of vaccinations and increased susceptibility to viruses in older adults are classical examples of the effects of aging on the immune system [3, 10, 11]. These effects are usually attributed to immunosenescence, a process that leads to changes in all immune cells and an inability to mount productive responses against pathogens and vaccinations. Cellular senescence, now termed replicative senescence, was originally defined as the loss of proliferative ability in replication-competent cells. Replicative senescence is thought to be a protective mechanism, designed to prevent stressed cells from undergoing malignant transformation [12]. The signature proteins that are upregulated that maintain senescence are the cyclin-dependent kinase inhibitors p21^WAF1/Cip1^ and p16^INK4a^ [13]. Senescent cells are increased in many different tissues with age, including the CNS [14–17]. The presence of senescent cells in the CNS may alter neuropathology in neurodegenerative disease, as clearance of senescent cells from the CNS was found to be beneficial in a mouse model of tauopathy [14].

Senescent cells also share a senescence-associated secretory phenotype (SASP). This includes pro-inflammatory cytokines, growth modulators, and chemotactic proteins. Interleukin-6 (IL-6), a pro-inflammatory cytokine, is perhaps the cytokine most associated with SASP. IL-8/C-X-C motif chemokine ligand 8 (CXCL8), a chemokine that recruits cells expressing C-X-C motif chemokine receptor 1 (CXCR1) as well as CXCR2 (often neutrophils), is also a key component of SASP. Immune cells produce many of the same molecules as seen in SASP in response to infections. These responses are designed to target damaged cells and pathogens and are normally robust, acute, and often self-limiting. This protective response is very much in contrast with chronic inflammation which tends to be long-lasting, and the host inflammatory response is responsible for tissue damage [18]. This low level, chronic inflammation which often occurs with aging and senescence has been termed inflammaging [19]. Because of the production of pro-inflammatory cytokines and chemokines that occurs with inflammation, the theory that anti-inflammatory treatments will be beneficial for diseases of aging has arisen [20]. But the pro-inflammatory cytokines may indicate a senescent immune response that has reduced functionality in other activities, such as phagocytosis [21]. In this case, either removing the senescent cells or pushing the cell back towards a robust acute response might be a better approach.

## Alzheimer’s disease

AD is a progressive neurodegenerative disease and the most common cause of dementia. The condition can develop undetected for years until the clinical manifestation of cognitive impairment and memory issues appear, which then progressively worsen over time [22]. Physiological changes in the brain begin to occur years before the onset of symptoms [23, 24]. The symptoms of dementia cause deterioration of an individual’s independence, which dramatically impacts the daily lives of patients and their families. There are currently no viable therapeutic options to treat the cognitive symptoms of AD. Clinical trials for new drugs have not seen significant results in alleviating the disease [25]. Although recently approved drugs have been found to clear pathology, they struggle to improve cognitive outcomes [26]. This disconnect between the pathology and cognitive impairment indicates an unknown factor we have not accounted for thus far. A possible reason for the lack of treatment options in AD may be the divergence in AD pathology and cognitive impairment that has been described in the literature. Studies such as Boyle et al. have uncovered evidence that the known neuropathology of AD cannot fully account for dementia seen in these elderly patients. The majority of variation in cognitive decline in these patients remains unexplained [27].

Clinical presentations suggestive of Alzheimer’s dementia are classified by assessments of cognitive status. However, AD continues to be biologically defined by neuropathological hallmarks [28]. This pathology is characterized by aberrant extracellular amyloid-beta (Aβ) aggregates, which form diffuse and neuritic plaques, and hyperphosphorylated tau aggregates, which form intraneuronal neurofibrillary tangles [29]. These pathologies are progressively accompanied by loss of synapses, neuronal death, and gross brain atrophy [29]. In addition to examining the known hallmarks of AD, there has been a resurgence in examining the non-neuronal cells of the CNS in the AD brain. When Alois Alzheimer first characterized brains with AD, he described glial cells with abnormal morphology, which we now know to be microglia and astrocytes [30–33].

## Parkinson’s disease

PD is the second most common neurodegenerative disorder affecting the elderly after AD [34, 35]. It is the most prevalent movement disorder, affecting over one million Americans and over four million individuals worldwide, and its incidence is expected to double by 2030 [36]. PD is a neurologic disease characterized by motor symptoms including tremors, rigidity, and postural instability. The clinical motor symptoms, such as shaking, rigidity, bradykinesia, and difficulty with walking and gait, presumably result from the accumulation of pathological processes that overwhelm the brain’s capacity to tolerate or compensate for their adverse effects. It is becoming clearer that the PD motor symptoms may only develop after years of ongoing neurodegenerative cell loss in the substantia nigra [37, 38].

The defining neuropathological features of PD are the loss of dopaminergic neurons in the substantia nigra and aggregation of alpha-synuclein protein, encoded by the *SNCA* gene, within neurons. Missense mutations in the *SNCA* gene as well as overproduction of wild-type alpha-synuclein can cause PD [39, 40]. Further reports found alpha-synuclein neuronal proteins present within Lewy bodies [41]. Lewy bodies are intracellular protein aggregates comprised mostly of alpha-synuclein, ubiquitin, and neurofilament, and their presence in neurons is a hallmark of PD pathology [42]. They are associated with activated microglia and dopaminergic neuron death [43, 44]. Indeed, alpha-synuclein induces microglial activation and morphological changes [45].

Parkinson’s research pioneer Arvid Carlsson first discovered that reduced dopamine levels caused PD-like symptoms [46], and subsequently proposed increasing dopamine levels through therapeutic intervention [47]. The dopamine precursor levodopa has since been successfully used to treat motor symptoms of PD. It may also be beneficial for cognitive decline in individuals with PD [48]. However, levodopa is not without its side effects, and there is a lack of effective strategies for the treatment of PD motor, cognitive, and behavioral symptoms beyond levodopa.

## Microglia

In both AD and PD, there are indications from pathology, genetics, and murine studies that CNS-resident immune cells play an important role in disease pathogenesis. Several tissues have their own specialized, resident macrophage with generic innate immune functions as well as tissue-specific roles [49]. Microglia serve as the resident immune cell of the CNS. While once highly debated, it is now accepted that microglia originate from hematopoietic progenitors in the yolk sac and emigrate to the CNS before the development of the brain, in mice that are embryonic day 8.5–10.5 [50–53]. Microglia detect and react to any nearby pathological agents. They constantly survey the environment, respond to injury and pathogens, and perform tissue repair [54–56]. Microglia closely interact with neurons and impact neuronal function, as they partake in neurogenesis and synaptic pruning [54, 56, 57].

Dystrophic microglia are a morphologically described subset of microglia that appear to have fragmentation of their branches and beading in their processes [58, 59]. Interestingly, the number of microglia with a dystrophic morphology was found to be greater in cases with either Alzheimer’s disease, dementia with Lewy bodies, or limbic predominant age-related TDP-43 encephalopathy compared to age-matched controls [60]. It has been postulated that the dystrophic morphology of microglia represents senescent microglia [58]. Senescent microglia exhibit reduced phagocytic and migration capabilities in comparison to activated microglia [61]. Markers of senescence were found to be increased in microglia in patients with AD [62]. These deviations in microglia homeostasis may contribute to the pathology observed in AD and PD. The murine tauopathy model, MAPT P301S PS19 mice, exhibits an increased population of microglia and astrocytes expressing the cyclin-dependent kinase inhibitor p16^INK4A^, which is a marker of senescence. Interestingly, removal of these cells leads to a decrease of hyperphosphorylation of tau and decreased degeneration of cortical and hippocampal neurons [14]. It is possible that depleting the pool of senescent glial cells may similarly be a novel therapeutic approach to alleviate neuropathological progression in AD and PD.

As both activated microglia and senescent microglia produce inflammatory molecules, it is important to distinguish between the functionality and pathogenicity of activated versus senescent microglia. Microglial activation and senescence may both arise from inflammatory insults, as repeated LPS stimulation has been shown to induce senescence in the mouse microglial cell line BV2 [63]. It was previously thought that in the context of neurodegenerative disease, microglia were inappropriately activated, and accordingly, returning them to a homeostatic state would be protective. However, if inflammatory microglial signals in these disease contexts are really from senescent microglia rather than activated microglia, then either eliminating senescent cells or restoring them to a responsive and plastic state would be beneficial. A greater understanding of the activated and senescent microglial phenotypes is imperative for progress in neurodegenerative disease research.

## AD microglia

**Fig. 1:**
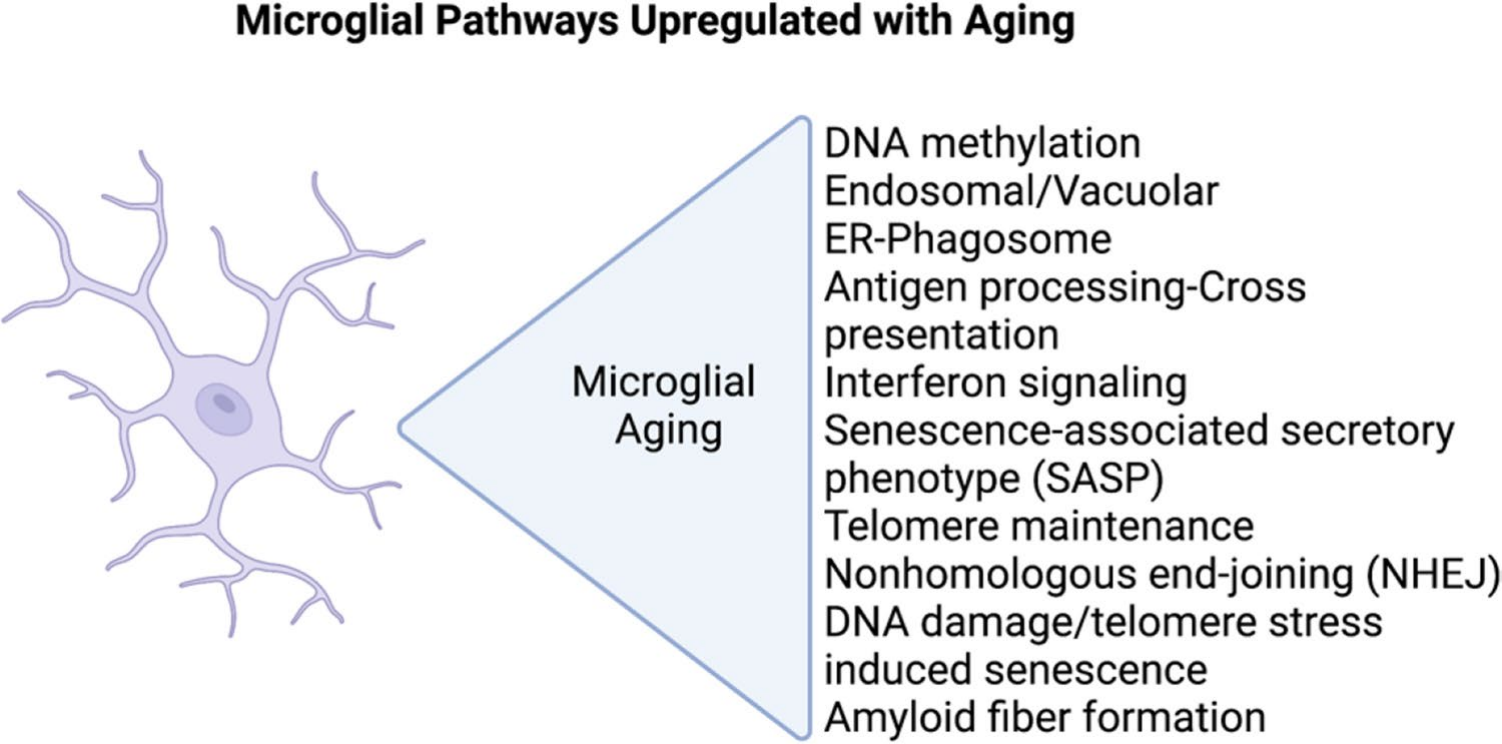
Pathways enriched in aged microglia. Two hundred seventy-one genes that were enriched in aged microglia were used to identify pathways that are upregulated in aged human microglia compared to microglia from younger individuals [72]. The figure was made in BioRender

After decades of research focused on neurons, genome-wide association studies (GWAS) have unveiled significant genetic risk for AD in innate immunity/microglia [64–66]. Many of these risk genes appear to be involved in phagocytosis. For example, *CD33*, *TREM2*, *ABI3*, *INPP5D*, and *PLCG2* have all been demonstrated to influence microglia phagocytosis of amyloid-beta or amyloid-beta deposition in the brain [67–70]. In addition to genetics, there have now been several studies that have examined postmortem microglia transcriptomics from aged individuals with or without AD. The microglia from AD patients appear to have an enhanced aging phenotype [71]. Aging microglia have altered expression of genes involved in cell adhesion and actin cytoskeleton dynamics, which suggests a functional decrease in cell motility [72]. Several of the pathways linked to aged microglia are suggestive of a senescent phenotype (Fig. 1). Importantly, genes that were found to vary with aging had very little overlap with genes of aging in murine microglia, emphasizing the importance of studying microglial aging in the human system [73].

Among the genes increased with aging across various studies is *IL15*. IL-15 is pro-inflammatory and helpful for a productive response against infections. It is also one of the cytokines that are secreted by senescent cells and is considered a part of SASP [74, 75]. Inhibition of IL-15 activity disrupts microglial activation and decreases cytokine and chemokine release [76]. IL-15/interleukin 15 receptor subunit alpha (IL-15RA) signaling may be neuroprotective, as IL-15RA knockout mice have increased motor neuron death after facial nerve axotomy [77]. IL-15 has wide-reaching effects on neural signaling in the brain, as it is thought to be essential in maintaining neurochemical homeostasis and is even thought to have anti-depressive effects on mouse behavior [78]. Additionally, IL-15 holds a very important immunological role in the development of natural killer cells and memory CD8 + T cells.

Another microglial gene that is increased in aging and AD is APOE. APOE has three main isoforms, named APOE e2, e3, and e4. Individuals having the APOE e4 isoform are at higher risk for LOAD [79]. Conversely, the APOE e2 haplotype is protective for AD and is associated with a decrease in the aging microglia phenotype [73]. APOE is a multifunctional protein with an important role in lipid transport [80]. Expression of APOE is upregulated early and implicated in the switch from homeostatic to neurodegenerative disease–associated microglia [81, 82]. While astrocytes are the main cell type for APOE production in the CNS, a recent mouse study suggested that microglial-produced APOE is important for synapse maintenance [83].

## PD microglia

Alpha-synuclein aggregates are a hallmark of PD [84], and microglia help clear and degrade misfolded alpha-synuclein [85, 86]. The activation state of microglia modulates the rate of protein degradation, with LPS-activated microglia showing decreased alpha-synuclein degradation and increased cytoplasmic accumulation [86]. And while microglial phagocytosis of extracellular alpha-synuclein can lead to degradation, microglia can also release alpha-synuclein through exosomes. Exosomes from these microglia can transport alpha-synuclein to neurons and induce protein aggregation in the neurons [87]. This mechanism has also been proposed for microglial-mediated transport of tau in AD [88]. These studies indicate a complex role for microglia in both clearing and transferring alpha-synuclein pathology in PD.

Alpha-synuclein has repeatedly been shown to activate both murine and human microglia [87]. In vitro experiments using the murine microglial BV2 cell line as well as in vivo experiments in mice demonstrate that exosomes carrying alpha-synuclein derived from PD patients enter microglia and induce activation, leading to enhanced microglial cytokine release and NO production [89]. Evidence of microglial activation has been found in the substantia nigra of postmortem PD brains, and in the 1-methyl-4-phenyl-1,2,3,6-tetrahydropyridine (MPTP)–induced mouse model of PD [90, 91]. Activated microglia in the PD brain exhibit increased expression of ICAM-1, a pro-inflammatory intercellular adhesion molecule [92]. These microglia also express the cytokines TNF-alpha and IL-6 [92, 93]. In addition to these pro-inflammatory markers, McGeer et al. described an increase of HLA-DR positive cells in the substantia nigra of patients with PD [94]. HLA-DR, part of the antigen-presenting machinery used to activate T cells, is almost exclusively expressed on innate immune cells, and therefore, in the CNS is specifically expressed in microglia. The human leukocyte antigen (*HLA*) locus is also genetically associated as discussed below.

## Genetics

The advent of unbiased genome-wide association studies has been critical in shifting the focus in neurodegenerative research away from a concentration on only neurons to one that includes other CNS cell types, specifically microglia. Many genes that have been associated with susceptibility to AD are significantly enriched in microglia compared to total brain tissue [73]. Risk genes for PD do not implicate microglia as clearly, as they are expressed in many CNS cell types. However, some of these genes are enriched in microglia, including the *HLA* region and *CTSB*. Understanding how one’s genetic background influences microglia behavior in neurodegenerative diseases will lead to a more comprehensive understanding of the disease mechanism, as well as identify potential therapeutic directions.

From work translating genetic associations to functional outcomes, it is becoming clearer that many of the genetic variants associated with LOAD lead to a hypofunctional innate immune system, specifically microglia [95]. For example, the AD-protective allele in the *PLCG2* gene leads to a proline to arginine amino acid change, and this mutation results in enhanced immune signaling [96, 97]. The AD-risk alleles in the *CD33*, *IL34*, *PILRA*, and *SPI1* loci lead to a dampened immune response [67, 68, 98–100]. Understanding the functional outcomes of these genetic associations has helped to reframe thinking about the approach to targeting the immune system in neurodegenerative disease. The previous thinking that the innate immune system needed to be suppressed is not supported by the genetic findings [20]. We hypothesize that aging, which leads to increased susceptibility to AD and PD, also induces a hypofunctional innate immune system and this, compounded with genetic risk factors, leads to a failure of microglia and the progression of AD.

The genetics of PD are not as clear in implicating innate immunity in the susceptibility to disease. Many of the genetic risk alleles in AD lead to a suppressed immune response, while some of the immune genetic hits in PD modify the pathways of antigen processing and presentation in immune cells. For example, *CTSB*, which is genetically linked to PD, codes for a lysosomal protease enriched in microglia, which is known to be important in processing antigens, including alpha-synuclein, for presentation by antigen-presenting cells to T cells [101, 102] [103]. Moreover, *CTSB* has been shown to influence the polarization of T cells through modulation of antigen-presenting cell cytokine production [104]. While we do not know about microglia specifically, the genetic association with PD in the *CTSB* locus leads to reduced expression levels in multiple tissues [103].

In addition to *CTSB*, other PD risk genes, *LRRK2*, *GBA*, and *BAG-3*, have all been implicated in antigen presentation as well [105–107]. We have shown that the common genetic association in the *LRRK2* locus leads to increased expression of *LRRK2* with the risk allele in a model of human microglia [108]. LRRK2 KO mice have innate immune cells that produce more proinflammatory cytokines upon activation and greater T cell proliferation in an antigen presentation assay [105], suggesting that LRRK2 expression may inhibit productive antigen presentation and activation of T cells. Whether the genetic variation changes the spectrum of antigens presented in the CNS of individuals with PD, and how this variation influences T cell phenotypes, is unknown.

The *HLA* complex region is an immune-specific, genetically associated locus for both AD and PD [109–113]. The proteins encoded in the *HLA* region present peptides (antigens) to T cells to activate antigen-specific immune responses. *HLA* is the strongest genetic association for most autoimmune diseases, and the pairing of particular autoimmune risk *HLA* genes with specific antigens can lead to inappropriate immune activation and destruction of very specific, disease-defining cell types. The *HLA* association with AD and PD implies that antigen presentation by innate immune cells (including microglia) to T cells is an important part of the susceptibility to both diseases.

One PD-focused study used deep sequencing of the *HLA* region to identify amino acid changes in the protein coded for by *HLA-DRB1* as a genetic risk for PD [114]. *HLA-DRB1* codes for part of MHC class II, which is the molecule on antigen-presenting cells responsible for presenting antigens to CD4 + helper T cells. Interestingly, the authors found an interaction between the protective genetic association and a history of smoking, which is a known protective factor for PD [115]. The authors hypothesize that smoking may lead to post-translational modifications of proteins such as alpha-synuclein, which then changes the binding affinity to MHC class II depending on the genetically associated amino acid changes. In a large study dissecting the *HLA* association with AD, the group found independent MHC class I and class II associations, suggesting that antigen presentation to both helper CD4 + T cells and CD8 + cytotoxic T cells is important to the disease susceptibility [112].

## T cells in AD and PD

**Fig. 2:**
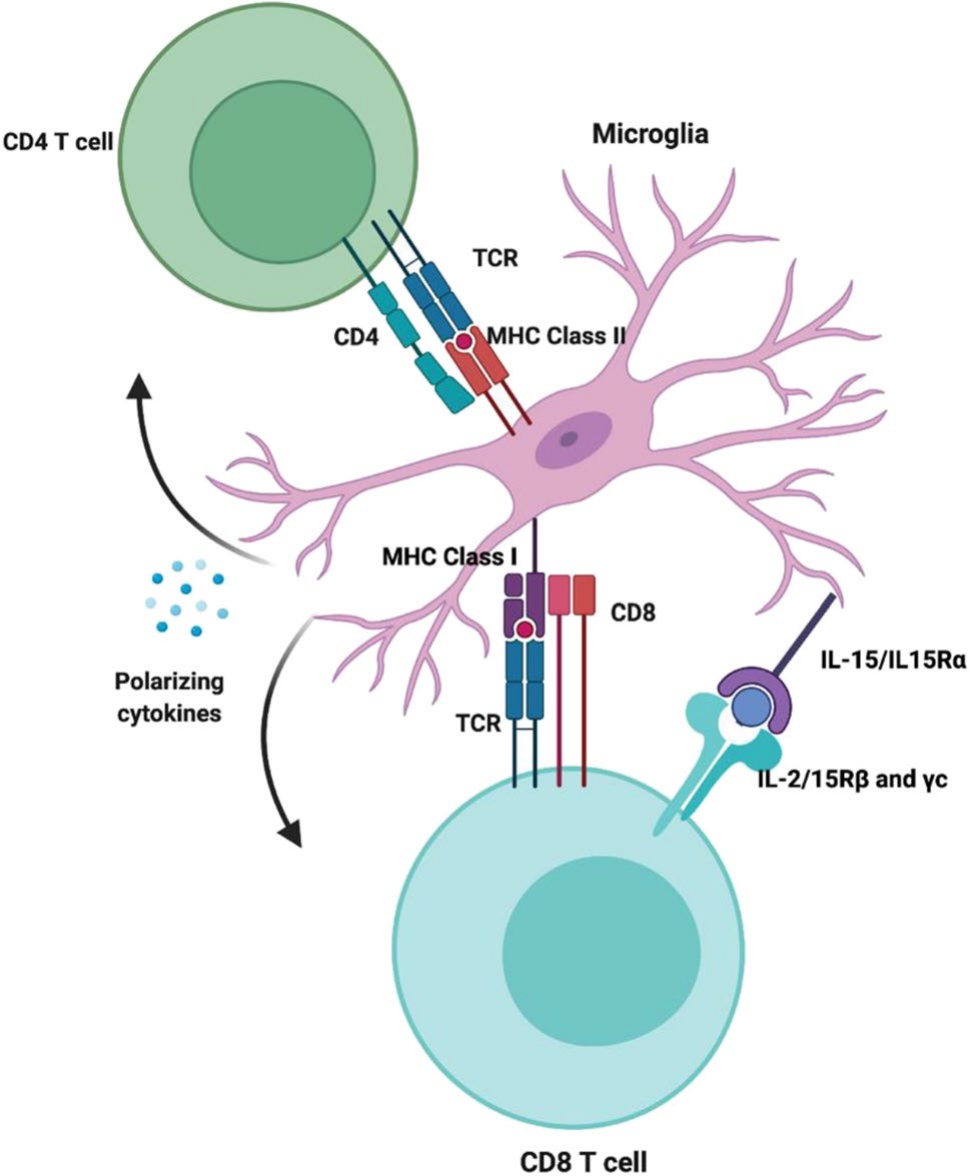
Microglia have antigen-presenting functions. Both CD4 + and CD8 + T cells infiltrate key CNS areas of neurodegeneration in AD and PD. Genetic risk for both diseases has been identified in the region that codes for the MHC proteins, which present antigens to T cells. Microglia also produce cytokines that can support the survival and polarization of T cells. Aged microglia produce more IL-15, an activating T cell cytokine. The figure was made in BioRender

Our understanding of microglial diversity in form and function is currently only rudimentary in the human system, especially their antigen-presenting function [116]. Microglia have the machinery to process and present antigens to both CD4 + and CD8 + T cells via MHC class I and II proteins (Fig. 2) [117, 118]. Infiltrating T cells have been described in the hippocampus in AD and the substantia nigra in PD, both primary foci of neurodegeneration in each disease respectively. Interestingly, in addition to T cell infiltration, these regions are also enriched with MHC class I and II expression in AD and PD. As previously described, the *HLA* region, which encodes MHC proteins, is genetically associated with AD and PD. In this way, microglia T cell interactions are implicated through several lines of evidence in the AD and PD brain [119–121]. A study looking at T cells in the CSF of PD and AD patients found clonally expanded CD8 + T cells in both diseases, suggesting that the T cells are infiltrating in response to a particular antigen [122]. In a study using the 5XFAD AD mouse model, intracerebroventricular injection of amyloid-beta-specific T cells induced MHC class II expression on microglia. These MHC class II–positive microglia have a neuroprotective phenotype with increased plaque-clearing abilities [123]. This highlights the reciprocal signaling between immune cells.

T cells are drastically affected by aging. Atrophy of the thymus with age is a clear example of this phenomenon and leads to a lack of new pluripotent T cells to repopulate the naïve T cell compartment [124]. The diversity of the T-cell receptor (TCR) repertoire found in 20–35-year-olds is reduced by 10–25% in 70–80-year-olds [125]. The phenotype of memory T cells in aging also changes, with an increase of clonality and T cells lacking the expression of necessary co-stimulatory molecules [126].

Peripheral immune aberrations, particularly in lymphocyte subsets, are abundant in PD patients. Specifically, it has been shown that peripheral T cells are diminished in PD patients [127, 128]. In AD, there is an increase in a particular type of CD8 + T cell that has been associated with chronic viral infections, and expansion of this T cell subset was found to be correlated with cognitive decline [122]. These effector memory T cells, which are defined by re-expression of the CD45RA protein, are termed T_EMRA_. These cells tend to be highly responsive to IL-15, which has been found to be increased in microglia in AD [129]. They have also been associated with a senescent phenotype; however, the senescent population is likely a subset of T_EMRA_ [130]. Interestingly, these T_EMRA_ T cells were found to be reduced in the circulation of individuals newly diagnosed with PD [131]. We have much work to do to understand the nuances of the role of various T cell populations in both AD and PD.

## Conclusions

A better understanding of the influence of the aging immune system on neurodegenerative diseases will be helpful in the search for novel therapeutic approaches. In terms of infiltrating T cells, it will be imperative to understand which populations are detrimental and which may be protective in disease contexts. For example, in one study, it was found that higher plasma IL-12p70 and IFNg in cognitively normal individuals was associated with reduced future cognitive decline [132]. IL-12p70 polarizes T cells towards the pro-inflammatory INFγ-producing T cell subset, suggesting that this subset of T cells may be protective. Defining how microglia act as antigen-presenting cells in key areas of neurodegeneration is also important to comprehensively understand their contribution to disease processes. The intersection of aging and genetics is also very likely to be critical to any immune dysregulation. The development of therapies that modify the role of these cells’ contribution to disease pathophysiology or enhance disease resistance will facilitate health and well-being among aging adults and markedly reduce health care expenditures.
